# Brain photobiomodulation: a potential treatment in Alzheimer’s and Parkinson’s diseases

**DOI:** 10.1016/j.tjpad.2025.100185

**Published:** 2025-04-25

**Authors:** Guillaume Blivet, Benjamin Touchon, Hugo Cavadore, Sara Guillemin, Frédéric Pain, Michael Weiner, Marwan Sabbagh, Cécile Moro, Jacques Touchon

**Affiliations:** a REGEnLIFE SAS, Montpellier, France; b CEA-Clinatec, Grenoble, France; c University of Montpellier, Montpellier, France; d RCTs, Lyon, France; e Université Paris-Saclay, Institut d'Optique Graduate School, CNRS, Laboratoire Charles Fabry, Palaiseau, France; f University of San Francisco, San Francisco, United States; g Barrow Neurological Institute, Phoenix AZ, United States

**Keywords:** Alzheimer, Dementia, Parkinson, Photobiomodulation, Treatment

## Abstract

Alzheimer’s Disease (AD) and Parkinson’s Disease (PD) are common neurodegenerative diseases, characterized by the progressive loss of synapses and neurons, leading to cognitive and motor decline. Their pathophysiology includes cerebral lesions, oxidative stress, neuroinflammation as well as brain-gut axis microbiota dysbiosis.

Preclinical investigations demonstrated that brain photobiomodulation (bPBM) reduces oxidative stress and inflammation, increases cerebral blood flow and enhance neurogenesis and synaptogenesis, which makes bPBM a promising treatment in AD and PD.

This review focuses on the clinical application of bPBM in AD and PD. It aims to provide a scientific overview of the current clinical knowledge, review recent clinical studies findings, and describe future directions and upcoming clinical studies.

So far, several clinical studies investigated bPBM therapy, at various parameters, both in patients with AD and related dementia, and PD. All demonstrate bPBM safety and bring valuable clinical information regarding efficacy, with particularly promising results in AD. However, their exploratory design and inconsistent quality lead to a low level of evidence, which currently does not support the widespread use of bPBM in clinical practice.

Future clinical research should address two gaps: the need for robust double-blinded RCTs vs sham with a higher number of patients and a longer follow-up, and the need for research focusing on dosimetry to determine which bPBM parameters are optimal. The ongoing or unpublished clinical studies on bPBM should fill in this gap.

## 1 Introduction

Alzheimer’s Disease (AD) and Parkinson’s Disease (PD) are common neurodegenerative diseases. Neurodegenerative diseases are characterized by the progressive loss of synapses and neurons, leading to cognitive and motor decline [[1], [2], [3], [4]].

AD, which is the most common form of dementia, is associated with progressive cognitive deficits as well as behavioral and mood disorders, leading to a loss of autonomy. Over 55 million people live with AD or other dementias worldwide, with forecasts reaching 78 million by 2030. AD and other dementias are the 7th leading cause of mortality worldwide and among the diseases with the highest cost to society [5]. The number of disability-adjusted life years they cause is estimated at 28.4 million [6].

PD is characterized by the progressive onset of motor symptoms (e.g., tremor, rigidity, akinesia) and non-motor symptoms (e.g., cognitive impairment, sleep disorders). Over 8.5 million people live with PD worldwide. Disability and death due to PD are increasing faster than for any other neurological disorder. The number of disability-adjusted life years due to PD is estimated at 5.8 million [7].

The pathogenesis of both AD and PD involves protein aggregates, leading to such diseases being considered to as proteinopathies, with AD being an amyloidopathy and PD being a synucleinopathy. Indeed, AD is characterized by the extraneuronal accumulation of β-amyloid proteins in the form of plaques and intraneuronal accumulation of hyperphosphorylated Tau proteins in the form of neurofibrillary tangles. These plaques and neurofibrillary tangles lead to progressive neuronal loss and synaptic degeneration [[1], [2], [3],^8^]. Of note, synaptic loss is a prominent neuropathological correlate of cognitive decline in AD [2]. On the other hand, PD is characterized by the aggregation of fibrillary deposits, mainly composed of misfolded α-synuclein proteins, within neurons (Lewy bodies) and neurites (Lewy neurites) [4,9]. Lewy bodies and neurites are associated with the death of dopaminergic neurons in the *substantia nigra pars compacta (SNpc),* as well as synaptic and axonal degeneration, ultimately leading to reduced dopamine levels [4,9]. While these protein aggregates are located in the cortex in AD (cortical lesions), they are located under the cortex in PD (subcortical lesions) [10]. Other elements described in the pathophysiology of AD and PD include mitochondrial dysfunction, oxidative stress, neuroinflammation as well as brain-gut axis microbiota dysbiosis [1]. However, their direct causal relationship to cognitive or motor symptoms of AD and PD is not fully established.

First therapies for AD and PD were only symptomatic [6,7]. In AD, the anti-amyloid monoclonal antibodies recently approved give hope of a therapy damping clinical decline [11,12]. Interventions directed at other aspects of AD than β-amyloid proteins (such as Tau proteins, neuroinflammation, oxidative stress) need to be investigated [13]. Until now, very few drugs targeting the gut microbiome have advanced through clinical trials process. The most advanced drug in that class is oligomannate [14], with quite controversial effects. In the years to come, treatment combinations will probably become the gold standard. To date, and despite extensive research, there are no disease-modifying treatments that would slow down PD progression.

Consequently, research continues and non-invasive brain stimulation treatments are currently being explored, including promising techniques such as brain photobiomodulation (bPBM) [15].

This review focuses on the clinical application of bPBM in AD and PD. It aims to provide a scientific overview of the current clinical knowledge, review recent clinical studies findings, and describe future directions and upcoming clinical studies. First, bPBM technology will be described. Then, recent clinical studies evaluating this technology in AD (and associated dementia) and PD will be reviewed and discussed. Finally, conclusions and future directions for clinical research on bPBM in AD and PD will be provided.

## 2 Brain photobiomodulation

### 2.1 Definition

PBM, previously known as low-level laser therapy, refers to the application of red (visible) to near-infrared (NIR) (invisible) (λ = 600–1100 nm) non-ionizing and non-thermal light over the body for therapeutic purposes [1,16].

### 2.2 Surface and targets

In bPBM, brain tissues are targeted, usually through non-invasive application of light over the surface of the head (transcranial application) [16]. Although more rarely used, other routes of administration exist, including non-invasive intranasal application or invasive intracranial application through brain implants [17]. However, most data on bPBM available in the literature involves transcranial application.

Transcranial bPBM bears limitations with respect to light penetration. Indeed, the light applied to the head must traverse multiple layers, including hair, scalp, blood, skull bone, and bone marrow, before reaching the brain. Each of these layers contributes to the attenuation of light intensity, which is a critical factor in the efficacy of bPBM [18]. Studies utilizing Monte Carlo simulations often model light diffusion through the skin and skull but typically do not account for the additional attenuation caused by hair. This omission is likely to result in an underestimation of the overall loss of light energy, as hair can significantly reduce light transmission. This is also why the forehead is a common and easier target due to the absence of hair [19]. Regarding penetration depth, NIR light has been shown to reach depths of up to 2–3 cm into brain tissue under optimal conditions [18]. However, this depth varies depending on factors such as wavelength, power density, and individual anatomical differences [20]. The targeted surface area on the head is also expected to impact the efficacy of transcranial bPBM, although the precise mechanisms of action are not fully understood [18,21,22]. Optimizing the location of light delivery could potentially enhance therapeutic outcomes.

Interestingly, remote PBM approaches have also demonstrated neuroprotective effects. For instance, Gordon et al. (2023) compared remote PBM targeting the abdomen or leg with transcranial PBM in mouse and non‐human primate models of PD, and found comparable degree of neuroprotection between groups [23]*.* The mechanisms behind remote PBM are not yet fully elucidated, but several theories have been proposed. One theory relates to the abscopal effect, mentioned for bPBM in the preclinical study by Blivet et al. (2018) [24]. Similar to the previously reported abscopal effect in oncology, PBM could induce systemic effects that impact distant tissues. Another theory pertains to brain-gut targets. Some studies combined bPBM and transabdominal PBM [[25], [26], [27]], to target the microbiome-gut-brain axis, which is crucial in the pathophysiology of both AD and PD [28]. This approach could leverage the brain-gut connection to amplify therapeutic effects. These remote approaches open new possibilities for PBM therapy, potentially overcoming the limitations of light penetration through the skull in transcranial bPBM.

### 2.3 Mechanism of action

Although bPBM mechanism of action remains to be fully elucidated, preclinical investigations suggest that bPBM primarily functions through the conversion of light energy into metabolic energy. According to this hypothesis, photons emitted from red to NIR light are absorbed by the Cytochrome C Oxidase (CCO), also known as the complex IV of the mitochondrial electron transport chain. This chromophore and enzyme, located in the inner membrane of mitochondria, plays a pivotal role in cellular respiration. The absorption of photons by CCO enhances its activity, leading to increased production of Adenosine TriPhosphate (ATP), the main energy-carrying molecule in cells. The upregulation of ATP production initiates several intracellular signaling pathways, resulting in the increase of intracellular calcium (Ca²⁺) and the activation of cyclic Adenosine MonoPhosphate (cAMP) production. These changes trigger downstream pathways which are crucial for cell survival and proliferation, such as the MAPK/ERK (Mitogen-Activated Protein Kinase/ Extracellular signal-Regulated Kinase), the PI3K/Akt (PhosphatidylInositol-3-Kinase / protein kinase B) and SIRT1 (Silent Information Regulator sirTuin 1) pathways. Overall, these molecular changes appear to result in several therapeutic effects : reduced oxidative stress, reduced inflammation, increased cerebral blood flow, enhanced neurogenesis and synaptogenesis [16,22,[29], [30], [31]].

More specifically, preclinical studies in AD models have shown that bPBM is associated with improved cognitive function, accelerated amyloid-beta (Aβ) degradation, reduced Aβ accumulation, and decreased microglial proliferation, which may contribute to mitigating neuroinflammation [31,32]. Additionally, bPBM has been associated with enhanced mitochondrial function in the neocortex and hippocampus. Preclinical studies in PD models have shown that bPBM is associated with improved locomotor activity (addressing characteristic motor symptoms), preservation of dopaminergic neurons in the SNpc and protection against mitochondrial structural damage [32]. Further studies are needed to establish clear causal links between these cellular and molecular changes and the observed improvements. Future research, including mechanistic studies and carefully designed preclinical and clinical studies, will be crucial in elucidating these relationships.

These observed effects make bPBM a promising treatment in AD and PD, since, in both diseases, oxidative stress and neuroinflammation may be responsible of cerebral function alteration. However, such potential therapeutic effects require emitted photons to cross the skull and meningeal barrier (down to the cortical layers in AD and beyond the cortex in PD). The literature reports that the penetration depth is maximal with a light wavelength around 810 nm [22,29]. It should be noted that the wavelength or source power are commonly mentioned parameters in PBM studies. Yet, solely, they do not define the dose delivery in terms of instantaneous local irradiance (in mW/mm^2^) 3D profile or integrated locally absorbed energy (in mJ/mm^3^). Among factors that define the light distribution and dose, we can distinguish between parameters of three different natures: those related to the device, those related to the PBM protocol and those related to the patient. The first two types of parameters are described later on in this review. Some studies investigating the effect of bPBM on functional connectivity suggest that bPBM could act on the default mode network (cortical areas that show synchronous activity when individuals are seemingly at rest, not engaged in any specific mental task [33]) and frontoparietal network (a brain region involved in cognitive control and executive function such task-switching and decision-making [34]). This is of particular interest since dysfunction in these networks are prominent features of AD and PD [16].

### 2.4 Device-related parameters

First, device parameters comprise stimulation parameters, i.e., location and type of stimulation (focal stimulation at a specific site near the skull or global stimulation on the whole skull) and stimulated surface area (cm^2^). Stimulation location should consider the targeted structure as well as physical issues such as light penetration and scattering. For example, intranasal stimulation was used to better target deep brain tissues [35,36] and extraoral stimulation of the gingival tissues was used to ease mucositis treatment [37]. Of note, details should be given on methods used to improve the light-source tissue interface (e.g., shaving, optical index adaptation, etc.). Regarding stimulation type, global stimulation with helmet or focal stimulation with direct fibered sources have been proposed to circumvent the limited penetration of a focal source due to light absorption and scattering [38]. It has also been shown that global stimulation alleviates the potentially detrimental thermal effects at the entrance points [39].

Device parameters also include physical parameters related to the light source itself. One of them is the source nature (laser diodes or Light Emitting Diodes [LEDs]). Usually, LEDs show an elliptical or round beam with moderate to strong divergence, while lasers provide a more collimated light. As such, while LEDs alleviate some of the safety issues related to laser light, laser is better suited for focal illumination with high irradiances. In optical physics, coherence describes the potential of two waves to interfere. LEDs are incoherent sources whereas lasers are coherent, with different degrees of coherence depending on the type and performances of the laser source (e.g., He-Ne, laser diodes, diode-pumped solid-state laser) [40]. Furthermore, laser light is usually polarized, with different degrees and types of polarization (linear, circular, elliptical) that may interact differently with the tissues, especially if tissue have birefringent properties such as collagen fibers [41]. Other intrinsic parameters of light source include the wavelength and spectral width (nm) or, even better, the full spectra, which can be easily measured with a fibered spectrophotometer. Indeed, the spectral distribution of the source plays a fundamental role both on PBM physics (light penetration and diffusion in the biological tissues varies strongly with the wavelength) and the underlying biological targets and associated PBM mechanisms [21,42].

Geometrical arrangement of the light source strongly shapes the light distribution and dosimetry in the brain tissues: the number of sources, the dimensions of the light emitters and the distances between the sources participate in defining the volume of the photomodulated tissues. The total power (mW), irradiance (mW/cm^2^) (i.e., power per surface unit) and spectral irradiance (i.e. irradiance per wavelength) (mW/cm^2^/nm) at the exit of the source are poorly informative. Indeed, the actual irradiance received by the tissues largely depends on geometric properties of the light beam such as the distance between the source and the tissue, the shape, uniformity and divergence of the beam, as well as the angle of the beam axis relative to the surface of the tissue [43]. It is thus good practice to provide the actual irradiance at the tissue entrance (mW/cm^2^), which can be measured using a calibrated power meter or a beam analyzer [44]. In addition, if fiber optics, optical guides or lenses, are used between the source and the tissues, their associated geometries, spectral transmission, numerical aperture and focal length should then be described.

Device-related parameters that are usually specified in clinical study publications are the number of sources and source nature. Most of the time, the geometrical arrangement of the light source, central wavelengths, wave emission mode and irradiance are also specified. However, parameters such as the light beam spectral and spatial distribution including divergence are rarely described.

### 2.5 Protocol-related parameters

Parameters related to therapeutic protocol encompass the number and frequency of sessions, time intervals between sessions, duration of each session, dose per session (J), duration of the overall therapy and cumulated dose (J). The cumulated dose corresponds to the total radiant energy deposited in the tissue. The number of sessions, duration of each session and dose per session have a direct influence on the cumulated dose. Of note, the dose per session (J) can be calculated from the irradiance (mW/cm^2^), stimulated surface area (cm^2^) and exposure time per session (min). In continuous mode, the exposure time corresponds to session duration while, in pulse mode, the exposure time can be deducted from the duty cycle, duration of a stimulus and number of stimulus.

The following protocol-related parameters are usually specified in clinical study publications: number of sessions, time intervals between sessions, duration of each session and duration of the overall therapy. However, and as highlighted by Fernandes et al. (2024), the stimulation protocol and parameters are not systematically totally reported [20].

### 2.6 Difference between brain photobiomodulation and visual flickering light stimulation

It should be noted that bPBM differs from visual flickering light stimulation which involves on-off flickering pattern light applied ocularly, usually at 40 Hz as this frequency induces gamma oscillation brain waves [45]. This emergent therapy relies on the discovery that several neurological disorders involve changes in gamma oscillations (20–50 Hz) [46]. However, recent studies in AD mice models have shown contradictory results: some demonstrated an effect on amyloid plaques or microglia morphology [46] while others did not [47]. It should be noted that, if bPBM uses red light (visible) in pulse mode, bPBM may comprise visual flickering light stimulation, potentially enhancing the therapeutic effect.

### 2.7 Dosimetry

So far, a vast range of bPBM parameters combinations have been tested and compared. However, a complete report of device-related and protocol-related parameters have seldomly be provided in publications. As a result, the PBM field suffers from conflicting results, resulting in confusion and lack of confidence in the technique as well as a lack of consensus about optimal parameters. To define which bPBM parameters are optimal, standardized methods to measure and report parameters are needed. Research focusing on dosimetry aims to define these standardized methods.

It should be noted that the characterization of dosimetry in bPBM is not straightforward. Most studies mention the cumulated dose (J), eventually over a given surface or volume of tissue (J/cm^2^ or J/cm^3^). However, it does not fully describe the immediate effects of light on tissues nor the protocol-related effects. Indeed, an identical dose can be obtained in a single session of 10 min, in 10 sessions of 1 min at the same light power, or in 1 session of 1 min at 10 times the power of the source. To characterize bPBM, it is important to also consider the instantaneous dose, or irradiance (mW/mm²), which indicates the power received by a unit of tissue surface at a given moment. This provides insight into the immediate biological effects of light, such as mitochondrial activation, and should help optimize treatment by ensuring that the light intensity is within a therapeutic range. While the cumulated dose gives a cumulative view of energy delivered, irradiance is crucial for understanding how the energy is distributed and how it impacts tissue at the cellular level during treatment. Both measures are useful for optimizing the therapeutic outcomes of bPBM.

The direct dose measurement of cumulated or instantaneous dose during bPBM presents significant technical and ethical challenges due to the need for invasive light detectors to implant locally. While this approach has been carried out in preclinical studies in rat for photodynamic therapy [48], its invasiveness strongly limits its implementation in humans, especially for non-invasive bPBM. Post-mortem measurements of NIR light penetration in human brain tissues [49] and skull [50] have been conducted. Yet the meaning of the brain tissue measurements is limited as dead tissues obviously behave differently in terms of light diffusion and absorption compared to living tissues. Recent studies indicate that light penetration and dose delivery through the skull depend on various patient-specific and protocol-specific parameters as discussed by Huang et al. (2024) [31].

To overcome these difficulties, the approach that has been followed in PBM (following studies in photodynamic therapy) is similar to what is currently done in clinical facilities for radiotherapy using ionizing radiations such as gamma-rays or protons. This involves creating treatment plans based on patient tissue geometry from anatomical imaging, followed by Monte Carlo simulations to calculate local deposited energy and evaluate different scenari. However, unlike well-characterized gamma-ray interactions with human tissues, optical methods face the additional challenge of accurately estimating optical tissue properties. This makes PBM dose estimation more complex compared to ionizing radiotherapies, where patient-specific data can be derived from X-ray images. Despite these challenges, this combined approach of post-mortem studies, preclinical research, and advanced simulations represents the current state of efforts to improve dose estimation and treatment planning in PBM, particularly for brain applications.

Although this goal is difficult to reach, there has been considerable effort to address the need for quantitative tools to efficiently simulate optical photon paths and the subsequent activated volume as well as thermal effects in 3D heterogeneous geometries. The reference method for the simulations of the photon path in tissues, is the Monte Carlo method. It is a statistical computational technique used to solve problems where no analytical solution can easily be obtained. In Monte Carlo simulations, photon packets are propagated and tracked within the tissues, along steps whose length and directions are defined by random sampling on probability distributions defined by the absorption and diffusion properties of the tissues [51]. The method is computer intensive as several millions of photons packets must be propagated (corresponding to hundreds of millions of random number sampling) to evaluate with a good statistical accuracy the light dose distribution in complex heterogeneous geometries. Since the first code simulating photon travel in tissue in semi-infinite layered geometries [52], Monte Carlo simulations have evolved to incorporate 3D anatomical meshed data from magnetic resonance imaging or computerized tomography in graphics processing unit accelerated geometries [53], heat diffusion effects [54,55] and end-users implementation [56]. Due to the difficulty to incorporate accurate optical properties for tissues, obtaining an absolute quantitation for patient-specific studies is still out of reach for optical methods. Thus, numerical simulations should not be intended to provide an absolute dose quantitation for treatment planning for example. However, the numerical simulations do provide (i) a good way to optimize the devices geometry considering relative doses obtained for different optodes [39] and (ii) a median estimation of the dosimetry for the targeted structure that can serve to set boundaries for the dose to be delivered [57].

In addition to being specified in clinical study publications, above-described bPBM parameters should be included in simulations carried out to evaluate the dose distribution for various devices or protocols. Disclosing such parameters would enable comparison across studies, therefore improving reproducibility.

## 3 Brain photobiomodulation in Alzheimer’s disease and associated dementia

### 3.1 Published clinical studies

At the time of this review, 9 clinical studies evaluating bPBM in AD (*n* = 3 studies), AD or dementia (*n* = 3), dementia (*n* = 2) and Mild Cognitive Impairment (MCI) (*n* = 1) were published, since 2017 (Table 1).

**Table 1:** Summary of published clinical studies on bPBM in AD, MCI or dementia.

Clinicaltrials.gov Reference	Study design	Study design details:	Patients	Intervention and PBM protocol parameters	PBM device parameters	Main results	Major limits
		Confirmatory / exploratory study Method to account for multiplicity of analyses (e.g. primary and secondary endpoints, hierarchized endpoints) Sample size calculation					
Berman et al. (2017) [59]	Double-blinded RCT (vs sham) *No information on sham device*	Exploratory No No	*N* = 11 (6 bPBM vs 3 sham, 2 withdrawals) Probable Alzheimer dementia (MMSE: 15–25)	bPBM (transcranial): Cognitolite (Maculume) 6-min treatment 28 sessions: 1/day for 28 days	LEDs Global stimulation (helmet) 1072 nm Pulse: 10 Hz, 50 % duty cycle *No information on irradiance and fluence*	After 28 days, in comparison to sham group: - No statistically significant difference - Trends in clock drawing, immediate recall, praxis memory, visual attention and task switching (TMT A&B) as well as electroencephalogram amplitude and connectivity measures	Low number of patients Lack of information on sham device No sham confirmation No clear comparison
Saltmarche et al. (2017) [35]	Case series	Exploratory No No	*N* = 5 Mild to moderate dementia or AD (MMSE: 10–24, mean: 17.4)	In-clinic treatment: bPBM (transcranial + intranasal): Vielight Neuro (Vielight Inc.) 20-min treatment 14 sessions: 2/week for 2 weeks, then 1/week for 10 weeks + Home treatment: bPBM (intranasal): Vielight 810 (Vielight Inc.) 25-min treatment 84 sessions: 1/day, except on days of in-clinic treatments, for 12 weeks	LEDs Focal stimulation (posterior transcranial, anterior transcranial and intranasal) 810 nm Pulse: 10 Hz, 50 % duty cycle Transcranial: 41 mW/cm^2^, 24.6 J/cm^2^ Intranasal: 23 mW/cm^2^, 13.8 J/cm^2^ Transcranial + intranasal: 309 J + LED Focal stimulation (intranasal) 810 nm Pulse: 10 Hz, 50 % duty cycle 14 mW/cm^2^, 10.65 J/cm^2^, 10.65 J	After 12 weeks: - Improved MMSE (*p* < 0.003) - Improved ADAS-cog (*p* < 0.023) - Positive effects reported by patients and families on function, sleep, angry outbursts, anxiety and wandering - No adverse event - Adherence to the home treatment protocol was high, as evidenced with the ‘‘Daily Home Treatment Journal’’ Precipitous declines were observed 4 weeks after treatment discontinuation	No control group Low number of patients
Chao et al. (2019) [63]	Open RCT (vs usual care)	Exploratory No No	*N* = 8 (4 bPBM vs 4 usual care) Dementia or AD (mean MMSE: 19.5 in bPBM group, 22.3 in usual care)	bPBM (transcranial + intranasal): Vielight Neuro Gamma (Vielight Inc.) 20-min treatment 36 sessions: 3/week for 12 weeks	LEDs Focal stimulation (posterior transcranial, anterior transcranial and intranasal) 810 nm Pulse: 40 Hz, 50 % duty cycle Posterior transcranial: 100 mW/cm^2^, 60 J/cm^2^ Anterior transcranial: 75 mW/cm^2^, 40 J/cm^2^ Intranasal: 25 mW/cm^2^, 15 J/cm^2^	After 12 weeks, in comparison to usual care group: - Different evolution of ADAS-cog (group x time interaction: F_1,6_ = 16.35, *p* = 0.007) - Different evolution of NPI frequency x severity total score (group x time interaction: F_1,6_ = 7.52, *p* = 0.03) - Different evolution of cerebral blood flow (group x time interaction: F_1,6_ = 8.46, *p* < 0.03), - No statistically significant difference in default mode network activity - No adverse effects - treatment diaries to measure adherence, but no published results	No sham No blindingLow number of patients
Nizamutdinov et al. (2021) [60]	Double-blinded RCT (vs sham) Sham device: designed identical to active device without emission of NIR light	Exploratory No No	*N* = 60 (40 bPBM vs 17 sham, 3 dropouts) Early- to mid-stage dementia or dementia-related symptoms (mean MMSE: 22.8 in bPBM group, 23.2 in sham group)	bPBM (transcranial + ocular): Cognitolite (Maculume) 6-min treatment 112 sessions: 2/day for 8 weeks	LEDs Global stimulation (helmet) 1060–1080 nm Continuous 23.1 mW/cm^2^, 8.3 J/cm^2^, 5 395 J	After 8 weeks: In bPBM group: - Improved MMSE (*p* < 0.001) - Improved in logical memory test - immediate recall (*p* < 0.05) - Improved TMT A&B time (*p* = 0.03) - Improved Boston naming test (*p* = 0.02) - Improved auditory verbal learning test - immediate recall (*p* = 0.002) - Improvement in auditory verbal learning test - delayed recall (*p* = 0.015) - No statistically significant difference in clock drawing test, clock copying test, logical memory test – delayed recall, digit span forward test, digit span backward test, Wechsler adult intelligence scale - revised digit symbol substitution test, word fluency test - Positive effects reported by patients on sleep, energy, daily living activities - Positive effects reported by caregivers on anxiety, mood, and positive daily routine - No adverse events - daily log to measure adherence, but no published results In the sham group: - No statistically significant difference	No comparison between groups No sham confirmation
Nagy et al. (2021) [62]	Open RCT (vs sham) *No information on sham device*	*Not specified* Yes (several primary endpoints (MoCA-B basic, quality of life – AD) but no hierarchization nor composite endpoint definition specified) Yes	*N* = 60 (30 bPBM vs 30 sham) AD (MoCA-B: 19–25, median: 23.96 in bPBM group, 23.42 in sham group)	bPBM (intranasal) + wrist PBM: Laspot watch 30-min treatment 72 sessions: 2/day 3 days/week for 12 weeks Associated with physical exercise	Laser Focal stimulation 650 nm *No information on wave emission mode, irradiance and fluence*	After 12 weeks, in comparison to sham: - Higher improvement in MoCA-B basic (*p* < 0.001) - Higher improvement in quality of life - AD (*p* < 0.001)	Lack of information on sham device No sham confirmation No blinding
Chan et al. (2021) [58]	Single-blinded RCT (vs sham) *Lack of information on sham device* However, according to Chan et al., “participants in the control group did not realize that they had a sham stimulation”	Exploratory No No	*N* = 18 (9 bPBM vs 9 sham) MCI *(no information on severity score)*	bPBM (transcranial): Wisefori 5–3800 (WiseforLtd) 350s-treatment 1 session	LEDs Focal stimulation (forehead) 810 nm Continuous 20 mW/cm^2^, 7 J/cm^2^	After 1 session, in comparison to sham: - Higher number of subjects who improved their visual memory (computerized Corsi block test) (*p* = 0.05) - Decrease of blood flow required to perform visual memory task in the bPBM group only (*p* = 0.008)	Single blinding Low number of patients
Kheradmand et al. (2022) [61]	Double-blinded RCT (vs sham) Sham device: identical to active device with laser diodes off	*Not specified* No No	*N* = 32 (16 bPBM vs 16 sham) Dementia (mean MMSE: 16 in bPBM group, 15.13 in sham group; mean CDR: 1.28 in bPBM group, 1.69 in sham group)	bPBM (transcranial): Model niltvirc102 (Noura Inst. Tehran) 10-min treatment 6 sessions: 3/week for 2 weeks	Laser Global stimulation (helmet) 630 + 810 nm Pulse: 75 Hz, 20 % duty cycle 90 mW/cm^2^, 56.5 J/cm^2^, 284.76 J	After 2 weeks, in comparison to sham: - Improvement in MMSE (*p* = 0.00005), persisting 6 weeks post-treatment (*p* = 0.000003) - No statistically significant difference in CDR	No sham confirmation Low number of patients
Blivet et al. (2022) [26]	Double-blinded RCT (vs sham) Sham device: identical to active device without NIR light and reduced red light by 10 % of the emission power	Exploratory Yes (primary endpoint: ADAS-cog) Yes	*N* = 53 (27 bPBM vs 26 sham) Mild to moderate AD (MMSE: 16–20; mean: 20.5 in bPBM group, 20.2 in sham group)	bPBM (transcranial) + transabdominal PBM: RGn530 (REGEnLIFE) 25-min treatment 40 sessions: 5/week for 8 weeks	Laser + LEDs Global stimulation (helmet + abdominal belt) 660 + 850 nm Pulse: 10 Hz, 50 % duty cycle Laser: 21.36 mW/cm^2^, 16.02 J/cm^2^ Infrared LEDs: 28.76 mW/cm^2^, 21.57 J/cm^2^ Red LEDs: 25.46 mW/cm^2^, 19.10 J/cm^2^ Trancranial: 12 866.3 J Transabdominal: 10 936.36 J	After 8 weeks, in comparison to sham: - Higher improvement of ADAS-cog comprehension subscore (*p* = 0.029) - Improved TMT B time (*p* = 0.012) - No statistically significant difference in ADAS-Cog (*p* = 0488), TMT A time, forward verbal span (*p* = 0.141) and backward verbal span 4 weeks after treatment discontinuation, in comparison to sham: - Improved forward verbal span (*p* = 0.033) - No statistically significant difference in incidence of adverse events (reported adverse effects were 3 headaches and 2 epistaxis) - Treatment compliance was thus defined as “very good” for 92 % of the patients (*N* = 46) and ‘poor’ for the remainder (8 %, *N* = 4). No significant differences were observed between the treatment groups.	No sham confirmation Reduced sample size and limited statistical power for some endpoints, resulting from premature ending of trial due to COVID-19
Chen et al. (2023) [64]	Open RCT (donepezil hydrochloride and bPBM vs donepezil hydrochloride)	Exploratory Yes (primary endpoint: *not specified*) No	*N* = 20 (10 bPBM vs 10 bPBM + donepezil hydrochloride) Mild to moderate AD (MMSE ≥ 10, mean: 18.5 in bPBM group vs 20.2 in bPBM + donepezil hydrochloride group)	bPBM (transcranial): *no information on brand name and manufacturer* 6-min treatments 120 sessions: 2/day 5 days/week for 12 weeks	LEDs Global stimulation (helmet) 800–820 + 1060–1080 nm Pulse: 10 Hz, 50 % duty cycle 5–35 mW/cm^2^, 0.9–6.3 J/cm^2^	After 12 weeks, in comparison to control: - Improved ADL (*p* = 0.0437) - Higher improvement in MMSE (*p* = 0.0253) - No statistically significant difference in ADAS-Cog (*p* = 0.5689), Clinician's Interview-Based Impression of Change plus caregiver input (CIBIC-plus), brain volume (*p* = 0.2048) and Geriatric Depression Scale (GDS) (*p* = 0.6450) - No statistically significant difference in incidence of treatment-emergent adverse events; 1 adverse effect (mild conjunctivitis) in bPBM group	No sham No blinding Low number of patients

Four (4) were double-blinded Randomized Controlled Trials (RCTs) vs sham, 1 was a single-blinded RCT vs sham, 3 were open RCTs (1 vs sham, 1 vs usual care and 1 combining bPBM and donepezil hydrochloride vs donepezil hydrochloride) and 1 was a case series. Of note, in the open RCT vs sham, both groups also underwent physical exercise. Some publications provide information regarding sham device: it consisted in device identical to active one without emitted light and/or with reduced emitted light. No studies surveyed healthcare professionals or patients in regard to sham device to confirm that the sham procedure did not break the blinding. Only Chan et al. (2021) mentioned that subjects did not realized they had a sham stimulation [58]. In terms of overall design, most studies were exploratory, with no defined method to account for multiplicity of analyses and no sample size calculation.

These studies included a total of 267 patients, with 5 to 60 patients included per study which illustrates the exploratory nature of these studies. Overall, 133 patients (49.8 %) had mild to moderate AD, 92 (34.4 %) dementia, 24 (9.0 %) dementia or AD and 18 (6.7 %) MCI. When reported, the mean Mini Mental State Examination (MMSE) score approximated 20 and the median Montreal Cognitive Assessment – Basic (MoCA-B) score approximated 23. Only Kheradmand et al. (2022) included more severe patients (mean MMSE approximating 15–16).

Out of these 267 patients, 147 were treated with active bPBM. Four (4) studies evaluated transcranial bPBM, 2 transcranial and intranasal bPBM, 1 transcranial and ocular bPBM, 1 intranasal bPBM and wrist PBM and 1 transcranial bPBM and transabdominal PBM. Stimulation was global in 5 studies (using a helmet); it was focal in the 4 others (targeting specific locations of the skull or being applied intranasally). The light source was LEDs in 5 studies, lasers in 2 and both LEDs and lasers in 1. Applied wavelengths ranged from 630 to 1080 nm, with 6 studies involving NIR light (around 810 nm and/or around 1070 nm), 1 involving red light (650 nm) and 2 involving both NIR and red light (630 and 810 nm). The wave emission mode was pulse mode in 5 studies, continuous mode in 2 and unknown in 1. In pulse mode, the most commonly used frequency was 10 Hz (with 50 % duty cycle); 40 Hz (with 50 % duty cycle) was used in 1 study and 75 Hz (with 50 % duty cycle) in another. The number of treatment sessions ranged from 1 to 120, with session duration ranging from 350 s to 30 min and session frequency from 1 per week to 2 per day. Overall therapy duration ranged from 1 day to 12 weeks.

All studies focused on cognitive function, as measured through various tools such as the MMSE or Alzheimer's Disease Assessment Scale cognitive subscale (ADAS-cog). Other endpoints were related to executive function (a subset of cognitive function), as measured through Trail Making Test parts A and B (TMT A&B), as well as to quality of life, activities of daily living, physiological parameters and safety.

Regarding safety, overall results support the safety of bPBM, with no or very few adverse effects and no statistically significant difference between the sham and the treated groups. Reported adverse effects were headaches, epistaxis and mild conjunctivitis.

Efficacy results are presented below per study type.

Among double-blinded RCTs vs sham, the study of Berman et al. (2021) did not evidence any statistically significant differences in cognitive functions between groups (transcranial bPBM vs sham) after 28 days of treatment. Only trends were observed in favor of active treatment, which could be due to the low number of patients (*n* = 11) [59]. In the study of Nizamutdinov et al. (2021), conducted in 60 patients, several statistically significant improvements were observed in scores related to cognitive functions after 8 weeks of treatment with transcranial and ocular bPBM but not with sham, but groups were not statistically compared [60]. Moreover, this study did not involve a method that takes into consideration multiplicity of analyses (e.g., definition of primary and secondary endpoints with conclusion on primary endpoint only, or definition of hierarchized endpoints with analysis of next endpoint only if the analysis of the previous one is significant). Kheradmand et al. (2022) (*n* = 32) found a statistically significant difference in MMSE score in favor of transcranial bPBM after 2 weeks (change: 2.31 ± 1.81 vs 0.13 ± 0.96, *p* = 0.00005), persisting 6 weeks post-treatment discontinuation (change: 2.53 ± 1.73 vs −0.25 ± 0.86, *p* = 0.000003), but no statistically significant difference in Clinical Dementia Rating (CDR) score was observed [61]. However, this study did not involve a method that takes into consideration multiplicity of analyses. Finally, Blivet et al.’s study evaluated transcranial bPBM and transabdominal PBM, in 53 patients. There was no statistically significant effect on the primary endpoint (ADAS-cog score). Statistically significant differences were observed in some secondary endpoints: ADAS-cog comprehension subscore (change: −0.4 ± 0.9 vs 0.3 ± 0.9, *p* = 0.029) and TMT B time (change: −45.8 ± 88 vs 53.1 ± 83.8 s, *p* = 0.012) after 8 weeks as well as forward verbal span score 4 weeks post-treatment discontinuation (change: 0.3 ± 0.8 vs −0.4 ± 1.1 s, *p* = 0.033). However, no differences were observed in other cognitive and executive endpoints. Of note, in this study, the treatment period was short (2 months) and statistical power reduced because of premature ending due to COVID-19 [26].

In the single-blinded RCT vs sham of Chan et al. (2021) which included 18 MCI patients, a statistically significant difference was observed in favor of transcranial bPBM treatment after 1 treatment session in terms of visual memory (subjects who showed improvement: 66.7 % vs 22.2 %, *p* = 0.05) [58]. However, this study did not involve a method that takes into consideration multiplicity of analyses.

Regarding open RCTs, in the study vs sham of Nagy et al. (2021) (*n* = 60), higher improvements were observed in intranasal bPBM and wrist PBM group in terms of MoCA-B score and quality of life - AD score, after 12 weeks (exact values not provided in the publication, *p* < 0.001) [62]. Both were primary endpoints; however, no hierarchization method description nor composite endpoint definition was identified in the publication. After 12 weeks as well, Chao et al. (2019) (*n* = 8) found statistically significant different evolutions between transcranial and intranasal bPBM vs usual care group in ADAS-cog (group x time interaction: F_1,6_ = 16.35, *p* = 0.007), NeuroPsychiatric Inventory (NPI) frequency x severity total score (group x time interaction: F_1,6_ = 7.52, *p* = 0.03) and cerebral blood flow (group x time interaction: F_1,6_ = 8.46, *p* < 0.03); no statistically significant difference was observed in default mode network activity [63]. However, this study did not involve a method that takes into consideration multiplicity of analyses. Chen et al. (2023) also found statistically significant differences between groups (donepezil hydrochloride and bPBM vs donepezil hydrochloride, *n* = 20) after 12 weeks in Activities of Daily Living (ADL) score (change: −3.6 ± 6.35 vs 3.1 ± 4.67, *p* = 0.0437) and MMSE (change: 4.4 ± 1.51 vs 1.0 ± 3.21, *p* = 0.0253) in favor of donepezil hydrochloride and bPBM group. No statistically significant differences were observed in other endpoints (ADAS-cog, impression of change, brain volume and depression) [64]. Although it is mentioned in the publication that the study had a defined primary endpoint, it is not specified whether this primary endpoint was ADL or MMSE.

Finally, in the case series of Saltmarche et al. (2017) (*n* = 5), cognitive function was improved after 12 weeks of treatment with transcranial and intranasal bPBM (change: MMSE: 2.60, *p* < 0.003; ADAS-cog: −6.73, *p* < 0.023) and positive effects on sleep and behavior were reported by patients and families [35]. Interestingly, precipitous declines were observed 4 weeks after treatment discontinuation.

### 3.2 Ongoing or unpublished clinical studies

Ten (10) clinical studies evaluating bPBM in AD, MCI or dementia, that were either ongoing or complete (but without associated publications identified) at the time we wrote this review, were identified on ClinicalTrials.gov. (Table 2).

**Table 2:** Summary of ongoing, or completed but unpublished, clinical studies on bPBM in AD, MCI or dementia of unknown etiology.

Clinicaltrials.gov reference	Study design	Study design details:	Patients	Intervention and PBM protocol parameters	PBM device parameters	Main evaluation criteria	Study completion date
		Confirmatory/ exploratory study Method to account for multiplicity of analyses (e.g. primary and secondary endpoints, hierarchized endpoints)					
NCT02537626	Double-blinded RCT (vs sham)	*Not specified* Yes (but several primary endpoints (ADAS-Cog and Alzheimer's disease cooperative study – activities of daily living) with no hierarchization nor composite endpoint definition specified)	*N* = 43 Mild to moderate AD (MMSE: 11–26)	bPBM (transcranial): ALS Laser (Erchonia) 10-min treatment 8 sessions: 2/week for 4 weeks	Laser Focal stimulation (frontal cortex, temporal regions, base of the skull) 640 nm *No information on wave emission mode, irradiance and fluence*	- ADAS-cog - Alzheimer's disease cooperative study – activities of daily living - MMSE - Subject’s study partner satisfaction with overall outcome rating	October 2020
NCT02851173	Single-blinded RCT (vs sham)	*Not specified* Yes (primary endpoint: psychomotor vigilance task)	*N* = 91 MCI *(no information on severity score)*	bPBM (transcranial): CG-5000 laser (Cell Gen Therapeutics) 8-min treatment (alternating every minute between two locations) 6 sessions: 1/week for 6 weeks	Laser Focal stimulation (two locations on right forehead) 1064 nm Continuous *No information on irradiance and fluence*	- Psychomotor vigilance task - Brain blood oxygen level dependent response to 2back task - 2back task correct responses - 2back task reaction time	April 2020
NCT03160027	Open RCT (vs usual care) (immediate treatment vs delayed treatment)	Exploratory Yes (but several primary endpoints (ADAS-Cog, clock-drawing test, default mode network functional connectivity, Arterial spin labeled perfusion magnetic resonance imaging measure and quality of life -AD) with no hierarchization nor composite endpoint definition specified)	*N* = 20 Dementia (MMSE>11)	bPBM (transcranial and intranasal): Vielight Neuro Gamma (Vielight Inc.) 20-min treatment. 42 sessions: 1/2days for 12 weeks	LEDs Focal stimulation (posterior transcranial, anterior transcranial and intranasal) *No information on wavelength* Pulse: 40 Hz, *no information on duty cycle* *No information on irradiance and fluence*	- ADAS-cog - Clock-drawing test - Default mode network functional connectivity - Arterial spin labeled perfusion magnetic resonance imaging measure - Quality of life -AD - Quality of life -AD from caregiver perspective - Caregiver burden inventory - NPI - Positive aspects of caregiving scale - Geriatric depression scale - short form in the caregivers	September 2020
NCT03405662	Double-blinded RCT (vs sham)	Exploratory Yes (primary endpoint: ADAS-cog)	*N* = 23 Mild to moderate AD (MMSE>13)	bPBM (transcranial and intranasal): Vielight Neuro Gamma (Vielight Inc.) 20-min treatment. 56 sessions: 1/2days for 16 weeks	LEDs Focal stimulation (posterior transcranial, anterior transcranial and intranasal) *No information on wavelength* Pulse: 40 Hz, *no information on duty cycle* *No information on irradiance and fluence*	- ADAS-cog - Color trail test - NPI - Alzheimer's disease cooperative study – activities of daily living - Change in plasma level of Aβ42 - Change in cerebrospinal fluid level of Aβ42 - Change in plasma level of Tau - Change in cerebrospinal fluid level of Tau - Change in plasma level of neurofilament light chain - Change in cerebrospinal fluid level of neurofilament light chain	January 2021
NCT03484143	Double-blinded RCT (vs sham)	Confirmatory Yes (but several primary endpoints (Severe Impairment Battery, Alzheimer's disease cooperative study – activities of daily living for severe AD) with no hierarchization nor composite endpoint definition specified)	*N* = 228 Moderate to severe AD (MMSE: 8–20)	bPBM (transcranial + intracranial): Vielight Neuro RX Gamma (Vielight Inc.) 20-min treatment 144 sessions: 6/week for 24 weeks	LEDs Focal stimulation (posterior transcranial, anterior transcranial and intranasal) 810 nm Pulse: 40 Hz, 50 % duty cycle *No information on irradiance and fluence*	- Severe Impairment Battery - Alzheimer's disease cooperative study – activities of daily living for severe AD - Euroquol – 5 dimensions - Quality of life - AD - NPI - Device-related adverse events	May 2023 Currently suspended due to slow recruitment
NCT03750409	Double-blinded RCT (vs sham)	*Not specified* Yes (but several primary endpoints (MMSE, ADAS-Cog and quantitative electro encephalography) with no hierarchization nor composite endpoint definition specified)	*N* = 100 Mild to moderate dementia	bPBM (transcranial): *no information on brand name and manufacturer* *No information on session duration* 112 sessions: 2/day for 8 weeks	*No information on source nature* Global stimulation (helmet) *No information on wavelength, wave emission mode, irradiance and fluence*	- MMSE - ADAS-Cog - Quantitative electro encephalography	October 2020
NCT04489082	Non-controlled prospective trial	*Not specified* Yes (but several primary endpoints (Beck depression inventory, Beck anxiety inventory, Quick dementia rating scale, brief pain inventory, global rating of change) with no hierarchization nor composite endpoint definition specified)	*N* = 400 Major depressive disorder or generalized or acute anxiety disorder or MCI or traumatic brain injury	bPBM (transcranial): *no information on brand name and manufacturer* 10-min treatment 5–6 sessions: 1/week for 5–6 weeks	Laser Focal stimulation (right prefrontal cortex) 1064 nm Continuous 250 mW/cm^2^, *no information on fluence*	In MCI patients: - Beck depression inventory - Beck anxiety inventory - Quick dementia rating scale - Brief pain inventory - Global rating of change - Patient depression questionnaire - Hamilton depression rating scale - Hamilton anxiety rating scale - Repeatable battery assessment of neuropsychological status - MoCA	December 2024
NCT04784416	Double-blinded RCT (vs sham)	*Not specified* Yes (primary endpoint: repeatable battery assessment of neuropsychological status)	*N* = 125 Amnestic MCI (CDR: 0.5–1.0)	bPBM (transcranial): *no information on brand name and manufacturer* 11-min treatment 24 sessions: 3/week for 8 weeks	Laser Focal stimulation (forehead, bilaterally) 808 nm Continuous 300 mW/cm^2^, *no information on fluence*	- Repeatable battery assessment of neuropsychological status - Addenbrooke's cognitive examination - Letter comparison test - Pattern comparison test - Stroop color and word test - TMT A&B - Face-name associative memory exam - Letter number sequencing - Change in Systemic Assessment for Treatment Emergent Events - Specific Inquiry	November 2025
NCT05563298	Single-blinded RCT (vs sham)	Exploratory Yes (but several primary endpoints (MMSE, California verbal learning test II, brief visuospatial memory test revised, TMTA&B) with no hierarchization nor composite endpoint definition specified)	*N* = 20 MCI due to AD (MoCA: 19–25)	bPBM (transcranial + intracranial): Vielight Neuro RX Gamma (Vielight Inc.) 20-min treatment 36 sessions: 6/week for 6 weeks	LEDs Focal stimulation (posterior transcranial, anterior transcranial and intranasal) 810 nm Pulse: 40 Hz, 50 % duty cycle *No information on irradiance and fluence*	- MMSE - California verbal learning test II - Brief visuospatial memory test revised - TMTA&B - Stroop Color and Word - Quality of life - AD - Blood lactate and lactate/pyruvate ratio - Structural and functional magnetic resonance imaging - Beck's depression inventory - Pittsburgh sleep quality index - Mild Behavioral Impairment Checklist	December 2023
NCT05926011	Double-blinded RCT (vs sham)	Confirmatory Yes (primary endpoint: ADAS-cog)	*N* = 108 Mild to moderate AD	bPBM (transcranial) + transabdominal PBM: RGn600 (REGEnLIFE) 20-min treatment 84 sessions: 5/week for 8 weeks, then 3/week for 8 weeks, then 2/week for 10 weeks	Laser + LEDs Global stimulation (helmet + abdominal belt) Pulse: 10 Hz, 50 % duty cycle	- ADAS-cog - MMSE - Computerized neurocognitive test - Digit symbol substitution test - TMT A&B - CDR - sum of boxes - AD composite score - Digit span test - Instrumental activities of daily living - Clinical global impression - Euroquol - 5 dimensions – 5 levels - Adverse device effects and device deficiencies - Blood markers	December 2025

Six (6) are double-blinded RCTs vs sham, 2 are single-blinded RCT vs sham, 1 is an open RCTs (1 vs usual care) and 1 is a non-controlled trial. Two (2) are confirmatory studies and 4 includes a method to account for multiplicity of analyses.

Overall, 1158 patients are included, or planned to be included, in these studies, from 20 to 400 patients per study. Overall, 422 patients (36.4 %) have AD, 216 (18.7 %) MCI and 120 (10.3 %) dementia. Among the 400 remaining patients (34.5 %), some have MCI (number not specified) and others have either major depressive disorder, generalized or acute anxiety disorder or traumatic brain injury. When mentioned, the MMSE score is comprised between 8 and 26, illustrating mild to moderate cognitive disorders.

Five (5) studies evaluate transcranial bPBM, 2 transcranial and intracranial bPBM and 2 transcranial and intranasal bPBM, 1 transcranial bPBM and transabdominal PBM. Stimulation is focal in 8 studies (targeting specific locations of the skull or being applied intranasally); it is global (using a helmet) in the 2 others. The light source is LEDs in 4 studies, lasers in 4, both LEDs and lasers in 1 and not specified in 1. When specified, applied wavelengths range from 660 to 1064 nm, with 5 studies involving NIR light (around 810 nm or 1064 nm), 1 involving red light (640 nm), 1 involving both NIR and red light (660 and 850 nm) (unspecified for the remaining 3 studies). The wave emission mode is pulse mode in 4 studies, continuous mode in 3 and unknown in 2. In pulse mode, the most commonly used frequency is 40 Hz (with 50 % duty cycle, when specified); 10 Hz (with 50 % duty cycle) was used in 1 study. The number of treatment sessions ranges from 5 to 144, with session duration ranging from 8 to 20 min and session frequency from 1 to 6 per week. Overall therapy duration ranges from 4 to 26 weeks.

Endpoints relate to cognitive function, neuropsychological status, quality of life, activities of daily living, caregiver burden, physiological and blood parameters as well as safety.

### 3.3 Key findings

Published clinical studies confirm the good safety of bPBM in AD, at the parameters used in these studies. Reported adverse effects were headaches, epistaxis and mild conjunctivitis.

Regarding efficacy, although most studies present promising results, their exploratory design and heterogeneous quality results in a low level of evidence, which does not enable to support the use of bPBM in current clinical practice yet.

The case series, open RCTs and single-blinded RCT reported statistically significant effects observed in cognition, psychobehavioral disorders as well as activities of daily living and quality of life. However, their design cannot rule out the placebo effect. In the double blinded RCTs vs sham, both statistically significant and non-significant results related to cognitive and executive endpoints were obtained. However, most of these studies remain exploratory, with no defined method to account for multiplicity of analyses and no sample size calculation.

Should the observed effects be related to bPBM, they could be the result of symptomatic action rather than disease-modifying action. Several arguments point in this direction. The first concerns the kinetics of effects appearance: effects rapidly appear - in a few weeks - after treatment beginning. The second concerns the kinetics of effects disappearance after treatment discontinuation. Results on this point are contradictory: Saltmarche et al. (2017) observed precipitous cognitive declines 4 weeks post-treatment discontinuation [35], whereas cognitive improvement was maintained 6 weeks post-treatment discontinuation in the study of Kheradmand et al. (2022) [61]. The last study to investigate residual effect, the one of Blivet et al. (2022) [26], seems to indicate an effect decrease after treatment discontinuation. A disease-modifying action would reduce neuronal loss or even progressively restore the neuronal pool. In the context of a slow-onset neurodegenerative disease such as AD, the effects of a disease-modifying treatment should, theoretically, be gradually established and maintained, at least for some time, after treatment discontinuation. The third argument concerns the identification of effects other than neuroprotective ones, such as increased cerebral blood flow [63], which could explain the cognitive effect, as well as its rapid appearance and disappearance. In particular, several studies in healthy subjects showed cognitive improvements, notably associated with increased cerebral perfusion [65,66]. A disease-modifying action of bPBM, in addition to a symptomatic action, cannot be ruled out, but does not appear to account for the short-term effects observed in these studies.

In conclusion, these studies bring valuable clinical information, which will be useful for larger, well-designed, confirmatory clinical studies. Over the past 4 years, the quality of studies increased, along with the number of included patients. There is now a need for robust double-blinded RCTs vs sham including a higher number of patients, allowing sufficient statistical power to conclude about the effect of bPBM.

The unpublished double-blinded RCTs vs sham, which plan to include up to 228 patients, should address this need. Considering their design and the number of patients planned to be included, they will enable to better evaluate the effect of bPBM on the whole range of AD symptoms. Some might also enable to differentiate symptomatic and disease-modifying effects and will provide information on effect persistence, as they comprise an evaluation post-treatment discontinuation (2 weeks in NCT02851173, 1 month in NCT04784416 and 26 weeks in NCT05926011). Finally, some aim to assess the impact of bPBM on blood and physiological parameters. These investigations, not carried out in previous studies, will provide additional valuable clinical information.

Regarding dosimetry, various device-related parameters and protocol-related parameters were used across clinical studies, as described above, and none of the studies compared these parameters. Therefore, it remains unknown which parameters are optimal for bPBM efficacy in AD.

## 4 Brain photobiomodulation in Parkinson’s disease

### 4.1 Published clinical studies

At the time of this review, 7 clinical studies evaluating bPBM in PD were published, since 2019 (Table 3).

**Table 3:** Summary of published clinical studies on bPBM in PD.

Reference	Study design	Study design details:	Patients	Intervention and PBM protocol parameters	PBM device parameters	Main results	Major limits
		Confirmatory / exploratory study Method to account for multiplicity of analyses (e.g. primary and secondary endpoints, hierarchized endpoints) Sample size calculation					
Hamilton et al. (2019) [74]	Case series	Exploratory No No	*N* = 6 PD	bPBM (transcranial for 5 patients, intranasal for 1 patient): Eliza (C & D Hamilton (authors of the publication)) 10–30 min 1–2/day, for 8 (*n* = 1), 12 (*n* = 2), 14 (*n* = 1) and 24 (*n* = 2) months	LEDs Global stimulation (helmet) for 5 patients, focal stimulation (intranasal) for 1 patient 670 + 810 nm for 4 patients treated transcranially, 670 + 850 nm for 1 patient treated transcranially, 660 nm for the patient treated intranasally Continuous	- 55 % of the initial signs and symptoms of the 6 patients showed overall improvement, 43 % stayed the same and 2 % got worse - Both motor and non-motor symptoms were affected; affected symptoms depended on the patient. - Changes slow in onset and sustained	No control group Low number of patients No use of standardized validated scales or scores
Santos et al. (2019) [67]	Double-blinded RCT (vs sham) Sham device: 5 s of active treatment followed by 55 s of inactive treatment (1/12th of energy used in active group)	Exploratory Yes (primary endpoint: MDS-UPDRS) No	*N* = 35 (17 active vs 18 sham) PD (HY: 1–2, mean: 1.5)	bPBM (transcranial): WARP 10 (Quantum Devices) 9-min treatment: 6 1-min blocks, alternating the LEDs between the right and left temples and with a 30–*sec* rest between blocks 18 sessions: 2/week for 9 weeks	LEDs Focal stimulation (right and left temples) 670 nm Continuous 60 mW/cm^2^, 8 J/cm^2^	After 9 weeks, in comparison to sham: - Higher decrease of TWMT - fast rhythm (*p* = 0.001) - No statistically significant differences in MDS-UPDRS III, SPES/SCOPA, static posturography, TMWT - preferred rhythm, TUG - Trend towards greater benefits in TUG (*p* = 0.094) - No adverse effects	Low number of patients Not a real sham
Liebert et al. (2021) [25]	Open waitlist-design randomized trial	Exploratory Yes (primary endpoint: TUG) No	*N* = 12 (6 patients immediately started treatment while 6 patients, who acted as their own controls, waited for 14 weeks before starting treatment) PD (HY: 1–3)	bPBM (transcranial + intranasal): Vielight Neuro Gamma (Vielight Inc.) 35-min treatment + Transabdominal and neck PBM: SYMBYX PDCare laser (SYMBYX Biome) 330-s treatment (30 s per targeted area) for in-clinic treatment / 660-s treatment (60 s per targeted area) for home treatment In-clinic treatment period: 24 sessions: 3/week for 4 weeks, then 2/week for 4 weeks, then 1/week for 4 weeks Home treatment period: 120 sessions: 3/week for 40 weeks for patients who immediately started treatment / 75 sessions: 3/week for 25 weeks for waitlisted patients	LEDs Focal stimulation (posterior transcranial, anterior transcranial and intranasal) 810 nm Pulse: 40 Hz, *no information on duty cycle* Transcranial + intranasal: 255 J + Laser (one less diode during home treatment) Focal 904 nm Neck+abdomen: 39.6 J Pulse: 50 Hz, *no information on duty cycle*	After 12 weeks, overall (both groups together): - Increase in TMWT - walk speed and - stride length (*p* < 0.01) - Decrease in TUG, - motor and - cognitive (*p* < 0.01) - Increase in step test - affected leg and s - unaffected leg (*p* < 0.01) - Increase in MoCA (*p* < 0.01) - Decrease in spiral test - dominant hand (*p* < 0.01) - Increase in tandem stance - affected leg behind (*p* < 0.05) - No improvement in 9-hole peg test, tandem stance - unaffected leg behind, single leg stance and micrographia After home treatment: - Sustained decrease in TUG and TUG - cognitive (*p* < 0.01) - Sustained increase in step test - affected leg and – unaffected leg (*p* < 0.01) - Sustained increase in MoCA (*p* < 0.01) - Sustained decrease in spiral test - dominant hand (*p* < 0.05) - Positive effects reported by patients or caregivers on mood, engagement and socialization - No adverse effects or safety concerns	No control group Low number of patients
Bicknell et al. (2022) [71]						After 12 weeks, overall (both groups together): - Decreased Firmicutes to Bacteroidetes ratio in most patients (75 %) - No significant changes in microbial diversity and taxa; a trend toward microbiome changes, including increases in some short chain fatty acids-producing bacteria, increases in genera recognized as beneficial to the microbiome and decreases in potential pathogens and some bacteria recognized as harmful to the microbiome.	
Bullock-Saxton et al. (2021) [68]	Double-blinded RCT (vs sham) *No information on sham device*	Exploratory No No	*N* = 20 (10 group 1 vs 10 group 2) + 2 lost to follow-up PD (HY: 1–3, mean: 2.4 in group 1, 2.4 in group 2)	bPBM (transcranial + intraoral): Mid-Laser 2.5 (Irradia) 330s: 66 s treatment per point 2 treatment phases of 4 weeks (separated by a 4-week washout period): - Group 1: 3/week sham during the first phase, 2/week active + 1/week sham during the second phase - Group 2: 3/week active during the first phase, 1/week active + 2/week sham during the second phase	Laser Focal stimulation (4 cranial points and 1 intraoral) 904 nm 42 J Pulse: 50 Hz, *no information on duty cycle*	When comparing the two groups: - No statistically significant differences in MoCA, 9-hole peg test, TUG, spiral test, dynamic step test - Minimum clinically important difference in 9-hole peg test after the second phase, in group 1 but not 2	Low number of patients No information on sham No sham confirmation
Hong et al. (2021) [72]	Non-controlled prospective trial	Exploratory Yes (primary endpoint: UPDRS) No	*N* = 18 PD (HY: 2–3)	bPBM (transcranial): Model-102 NIR (HYL) 30-min treatment 10 sessions: 5/week for 2 weeks Simultaneous administration with hydrogenated water	LEDs Focal stimulation (posterior transcranial) 904 nm 6 mW/cm^2^ *No information on wave emission mode*	After 2 weeks: - Improvement of UPDRS (*p* < 0.001), UPDRS part I (*p* < 0.001), UPDRS part II (*p* < 0.01) and UPDRS part III (*p* < 0.05) - Improvement of UDPRS depression item (*p* < 0.05), falling (unrelated to freezing) item (*p* < 0.01) and rest tremor item (*p* < 0.05) - No change in blood test - No adverse events After therapy cessation for 1 week: - UPDRS part I still improved - UPDRS part II and III no longer improved	No control group Low number of subjects
Liebert et al. (2023) [73]	Non-controlled prospective trial Follow-up trial of the original trial reported by Liebert et al. (2021) [7]	Exploratory No No	*N* = 8 PD (HY: 1–3)	Continuation of the home treatment period up to 3 years: bPBM (transcranial): Coronet Duo (Well Red Pty Ltd) 12-min treatment 6/week up to 3 years + Transabdominal and neck PBM: SYMBYX PDCare laser (SYMBYX Biome) 660-s treatment (60 s per targeted area) 3/week up to 3 years	LEDs Global stimulation (helmet) 670 + 810 nm Pulse: 40 Hz, 12 + 20 % duty cycle + Laser Focal 904 nm Pulse: 50 Hz, *no information on duty cycle*	After 3 years, only descriptive results (no statistical analysis): - Improvement of median scores for MoCA, TMWT, step test, spiral test (*n* = 5) - No improvement of tandem stance and single leg stance (*n* = 5) - No device-related safety issues or adverse events - 7 participants reported using both PBM devices consistently since the 1-year assessment and 1 acknowledged to not using it consistently. 5 patients reported using PBM consistently after 2 and 3 years.	No control group Low number of subjects Inconsistent use of PBM devices by some patients
McGee et al. (2023) [69,70]	Double-blinded RCT (vs sham) Sham device: identical to active device without emitted light	*Not specified* Yes (several primary endpoints which seem to be hierarchized: safety, MDS-UPDRS-III) No	*N* = 40 (20 active vs 20 sham) PD (HY: 1–2)	bPBM (transcranial): SYMBYX Neuro (SYMBYX Biome) 24-min treatment 72 sessions: 6/week for 12 weeks	LEDs Global (helmet) 635 + 810 nm 1137 J *No information on wave emission mode*	After 12 weeks: - No statistically significant difference in MDS-UPDRS-III score and subscores - No safety concerns or adverse events, apart from occasional temporary and minor dizziness - adherence evaluation by the carers but no published results	No sham confirmation

Three (3) were double-blinded RCTs vs sham, 1 was an open waitlist-design randomized trial, 2 were non-controlled trials and 1 was a case series. Of note, in 1 non-controlled trial, treatment also involved hydrogenated water. Two (2) publications provide information regarding sham device: in one case, it consisted in a device identical to the active one without emitted light; in the other, it was not a real sham (5 s of active treatment followed by 55 s of inactive treatment). No studies surveyed healthcare professionals or patients in regard to sham device to confirm that the sham procedure did not break the blinding. In terms of overall design, most studies were exploratory, with no sample size calculation. Four (4) included a method to account for multiplicity of analyses (unique primary endpoint).

These studies included a total of 139 patients, with 6 to 40 patients included per study which illustrates the exploratory nature of these studies. Included patients displayed Hoehn and Yahr (HY) scores between 1 and 3, along with an unilateral or a bilateral disease involvement. Nonetheless, all patients were physically independent, without severe functional disability.

Out of these 139 patients, 101 were treated with active bPBM. Three (3) studies evaluated transcranial bPBM, 1 transcranial and intraoral bPBM, 1 transcranial and intranasal bPBM, 1 transcranial and intranasal bPBM as well as transabdominal and neck PBM, 1 transcranial bPBM as well as transabdominal and neck PBM. Stimulation was focal in 4 studies (targeting specific locations of the skull or being applied intranasally or intraorally), global (using a helmet) in 2 and both focal and global in 1. The light source was LEDs in 4 studies, lasers in 1 and both LEDs and lasers in 2. Applied wavelengths ranged from 660 to 940 nm, with 3 studies involving NIR light (810 and/or 904 nm), 1 involving red light (670 nm), 3 involving both NIR and red light (635 and 810 nm, or 670 and 810 nm). The wave emission mode was pulse mode in 3 studies, continuous mode in 2 and unknown in 2. In pulse mode, the used frequencies were 40 or 50 Hz (duty cycle only specified in 1 study). The number of treatment sessions ranged from 10 to hundreds or thousands of sessions in the case series, with session duration ranging from 330 s to 35 min and session frequency from 1 per week to 2 per day. Overall therapy duration ranged from 2 weeks to 24 months.

All studies focused on mobility and motor function. Other endpoints were related to cognition and other non-motor symptoms, microbiota, blood parameters and safety.

Regarding safety, overall results support the safety of bPBM, with no or very few device-related adverse events. Reported adverse effects were temporary and minor dizziness.

Efficacy results are presented below per study type.

Among double-blinded RCTs vs sham, the study of Santos et al. (2019) conducted in 35 patients treated for 9 weeks showed a statistically significant difference in favor of transcranial bPBM group in one secondary endpoint: Ten-Meter Walk Test (TMWT) - fast rhythm (change: −0.6 vs 0.0 s, *p* = 0.001). However, no statistically significant differences were observed in Movement Disorder Society (MDS) - Unified Parkinson's Disease Rating Scale (UPDRS) III which was the primary endpoint, nor other secondary endpoints (Short Parkinson's Evaluation Scale/SCales for Outcomes in PArkinson’s disease [SPES/SCOPA], static posturography, TMWT - preferred rhythm and Timed Up and Go test [TUG]), although there was a trend in TUG (change:−0.4 vs 0.2 s, *p* = 0.094) [67]. In the study of Bullock-Saxton et al. (2021), 20 patients were treated with transcranial and intraoral bPBM according to two different treatment patterns involving active and sham treatments over 12 weeks. No statistically significant differences between the groups were observed in scores related to cognition, fine motor skills, and mobility. After 12 weeks of treatment, McGee et al. (2022) (*n* = 40) found no statistically significant difference between groups (transcranial bPBM vs sham) in MDS-UPDRS III score and subscores [69,70].

In the open waitlist-design randomized trial reported by Liebert et al. (2021) and Bicknell et al. (2022) [71], 12 patients were treated with transcranial and intranasal PBM as well as transabdominal and neck PBM. After 12 weeks of in-clinic treatment, statistically significant improvements were observed in some scores related to mobility (median change: TMWT walk speed: 0.58 m/s, TMWT stride length: 0.15 m, TUG: −0.9 s, TUG motor: −1.0 s, TUG cognitive: −3.5 s, *p* < 0.01), balance (step test - affected leg: 4.5, *p* < 0.01; step test – unaffected leg: 3.5, *p* < 0.01; TS affected leg behind: 3.8, *p* < 0.05), cognition (MoCA: 2, *p* < 0.01) and fine motor skills (spiral test - dominant hand: −3.6, *p* < 0.01) but not all. After 25- or 40-week home treatment, there was a sustained improvement in some of these scores (median change: TUG: −1.42, *p* < 0.01; TUG cognitive: −0.9, *p* < 0.01; step test - affected leg: 5.5, *p* < 0.01; step test - unaffected leg: 7.5, *p* < 0.01; MoCA: 3.9, *p* < 0.01; spiral test – dominant hand: −7.5, *p* < 0.05). The primary endpoint corresponded to TUG [25]. Regarding microbiota, after the in-clinic treatment, the Firmicutes to Bacteroidetes ratio, often interpreted as a proxy for gut health, was decreased in most patients (75 %) (mean decrease: −3.02) but there were no significant changes in microbial diversity and taxa [71].

Regarding non-controlled trials, in the study of Hong et al. (2021) (*n* = 18), after 2-week transcranial bPBM and hydrogenated water treatment, statistically significant improvements were observed in UPDRS scores (UPDRS: *p* < 0.001; UPDRS I: 1.18 ± 1.78 vs 3.67 ± 3.77, *p* < 0.001; UPDRS II: 13.18 ± 7.08 vs 17.94 ± 8.21, *p* < 0.01; UPDRS III: 21.47 ± 11.60 vs 26.33 ± 13.28, *p* < 0.05), which was the primary endpoint, and UPDRS items (*p* < 0.01 or *p* < 0.05 depending on the item). No change was observed in blood parameters [72]. In the study of Liebert et al. (2023), 8 patients who previously participated to the double-blinded RCT reported by Liebert et al. (2021) [25] and Bicknell et al. (2022) [71] were treated for 3 additional years with transcranial bPBM treatment and transabdominal and neck PBM. Improvements in median scores related to cognition, mobility, dynamic balance and fine motor skills were observed but not statistically tested. No improvement was observed in static balance function [73].

Finally, in the case series of Hamilton et al. (2019), both motor and non-motor symptoms of PD were affected in the 6 patients treated with transcranial or intranasal bPBM for 8 to 24 months: 55 % of initial symptoms showed overall improvement, 43 % remained stable and 2 % got worse [74].

### 4.2 Ongoing or unpublished clinical studies

Five (5) clinical studies evaluating bPBM in PD, that were either ongoing or complete (but without associated publications identified) at the time we wrote this review, were identified on ClinicalTrials.gov (Table 4).

**Table 4:** Summary of ongoing, or completed but unpublished, clinical studies on bPBM in PD.

Clinicaltrials.gov reference	Study design	Study design details:	Patients	Intervention and PBM protocol parameters	PBM device parameters	Main evaluation criteria	Study completion date
		Confirmatory / exploratory study Method to account for multiplicity of analyses (e.g. primary and secondary endpoints, hierarchized endpoints)					
NCT03551392	Double-blinded RCT (vs sham)	Exploratory Yes (primary endpoint: ARENA)	*N* = 135 Healthy or PD (score HY: 0.5–3.5)	In-clinic treatment: bPBM (transcranial + intranasal): Medx Console System (Medx Lasers) 1.5 h 16 sessions: *no information on frequency of sessions,* for 12 weeks + Home treatment: bPBM (intranasal): Vielight 810 (Vielight Inc.) 25-min treatment 48 sessions: 4/week for 12 weeks	LEDs Focal stimulation (head and intranasal) *No information on wavelength* *No information on wave emission mode* + LED Focal stimulation (intranasal) 810 nm Pulse: 10 Hz, 50 % duty cycle	- ARENA, learning and memory - National Institutes of Health (NIH) examiner executive composite score - NIH toolbox emotion, negative affect scale - NIH examiner, verbal fluency domain the NIH examiner, working memory domain - NIH examiner, cognitive control domain - NIH toolbox emotion, psychological well-being scale	October 2024
NCT04261569	Open-label RCT (vs no intervention)	Exploratory Yes (primary endpoint: emergent adverse events)	*N* = 14 PD (HY: 1–2)	bPBM (intracranial): Ev-NIRT *(no information on manufacturer)* 1-min on and 5-min off, for 4 years	*No information on source nature* Focal stimulation (implant) 670 nm Pulse: 150 Hz, *no information on duty cycle*	- Emergent adverse events -Annual neuronal loss - MDS-UPDRS - Behavioral Evaluation in Parkinson's disease - Lille Apathy Rating Scale - Beck depression inventory - Non-motor symptoms scale - Substitution therapy - Parkinson disease quotiation-39 - Walking speed and parameters	April 2028
NCT05152706	Open-label RCT (aerobic exercise vs bPBM vs aerobic exercise + bPBM vs usual care)	*Not specified* Yes (primary endpoint: MDS-UPDRS)	*N* = 32 PD (HY: 1–2)	bPBM (transcranial): *no information on brand name and manufacturer* *No information on session duration* 48 sessions over 6 months, *no information on frequency of sessions* Associated with, or replaced by, aerobic exercise in some groups	Laser *No information on stimulation type* *No information on wavelength* *No information on wave emission mode*	- MDS-UPDRS - Disabilities of the arm, shoulder and hand - Static posturography - TMWT - TUG - Grip strength	June 2023
NCT05959772	Open-label (blinded outcomes assessor) RCT (vs sham)	*Not specified* Yes (primary endpoint: visual numeric scale)	*N* = 82 PD (HY: 3)	bPBM (transcranial): *no information on brand name* (Bright Photomedicine) 4- to 9-min treatment *No information on number and frequency of sessions;* treatment for 5 weeks	LEDs *No information on stimulation type* 850 nm *No information on wave emission mode*	- Visual numerical scale - McGuill pain questionnaire - King’s Parkison’s disease pain scale	June 2025
NCT06036433	Double-blinded crossover RCT (vs sham)	*Not specified* Yes (primary endpoint: TUG)	*N* = 60 Moderate PD (HY: 1–2)	bPBM (transcranial) SYMBYX Neuro v2.0 (SYMBYX Biome) + transabdominal PBM: SYMBYX PDCare laser (SYMBYX Biome) 30-min treatment 24 sessions: 3/week for 8 weeks Associated with exercise	LEDs Global stimulation (helmet) *No information on wavelength* *No information on wave emission mode*	- TUG - MDS UPDRS - TMWT - MoCA - 9-hole peg test - Spiral test - Writing test - Parkinson disease quotiation-39 - Parkinson's disease sleep scale - Smell test - Beck depression inventory - Beck anxiety inventory	May 2024

Two (2) are double-blinded RCTs vs sham and 3 are open RCTs (1 vs sham, 1 vs no intervention, 1 with various groups [aerobic exercise, bPBM, aerobic exercise and bPBM or usual care]). Two (2) remain exploratory studies (exploratory/confirmatory nature not specified for the remaining 3 studies). All studies include a method to account for multiplicity of analyses.

Overall, 323 patients are included, or planned to be included, in these studies, from 14 to 135 patients per study. Overall, the HY scale score is comprised between 0.5 and 3.5 (patients in the first stages of PD). Of note, 1 study enrolls both patients with PD and healthy volunteers.

Two (2) studies evaluate transcranial bPBM, 1 transcranial and intranasal bPBM, 1 intracranial bPBM and 1 transcranial bPBM and transabdominal PBM. Stimulation is focal in 2 studies (targeting specific locations of the skull or being applied intranasally), global (using a helmet) in 1 and not specified in 2. The light source is LEDs in 3 studies, laser in 1 and not specified in 1. When mentioned, applied wavelengths range from 670 to 850 nm, with 2 studies involving NIR light (810 or 850 nm), 1 involving red light (670 nm) (unspecified for the remaining study). The wave emission mode is pulse mode in 1 study (150 Hz, duty cycle not specified) and not specified in the 4 others. When specified, the number of treatment sessions ranges from 24 to 64, with session duration ranging from 4 min to 1.5 h and session frequency from 3 to 4 per week. Of note, these parameters do not apply to intracranial bPBM; in the study on intracranial bPBM, the implant functions for 1 min every 6 mins. Overall therapy duration ranges from 5 weeks to 4 years.

Endpoints relate to mobility, cognitive function, behavior / emotional function, quality of life and safety.

### 4.3 Key findings

Published clinical studies confirm the good safety of bPBM in PD, at the given parameters. Reported adverse effects were temporary and minor dizziness.

Regarding efficacy, bPBM does not seem to have an effect on PD motor function, evaluated through UPDRS III. Moreover, its effect in non-motor symptoms, including cognitive impairment, remains to be confirmed.

The case series, non-controlled trials and open waitlist-design randomized trial reported statistically significant effects on disease symptoms, cognition, mobility, balance and fine motor skills endpoints. However, their design cannot rule out the placebo effect. In the 3 double blinded RCTs vs sham (all conducted in a limited number of patients), no statistically significant results were found for most endpoints, including UPDRS III. Of note, most studies were exploratory, with no sample size calculation.

Most studies which evaluated non-motor symptoms did so through an informal evaluation (apart for cognition). Therefore, the effect of bPBM on these symptoms, particularly neuropsychiatric ones, remains to be further explored, especially in view of their impact on quality of life - which is at least as important as motor symptoms’ impact. Should it be confirmed, bPBM effect on cognitive impairment could be anticipated given the positive results obtained on cognitive function in patients with AD [26,35,58,[60], [61], [62]] and healthy subjects [65,66]. Of note, none of the studies in PD enable to differentiate the symptomatic effect from the disease-modifying effect.

In conclusion - and as for clinical studies in AD and associated dementia - these studies bring valuable clinical information regarding bPBM safety and efficacy in PD. There is now a need for double-blinded RCTs vs sham including a higher number of patients, allowing a sufficient statistical power to further investigate PD non-motor symptoms, including cognition.

The 2 unpublished double-blinded RCTs vs sham, which respectively plan to include 60 and 135 patients, should address this need. One of them (NCT04261569) is of particular interest as it will enable to evaluate the disease-modifying effect due to its long treatment period (4 years).

Regarding dosimetry - and as for clinical studies in AD and associated dementia - various device-related parameters and protocol-related parameters were used across clinical studies, as described above, and none of the studies compared these parameters. Therefore, it remains unknown which parameters are optimal for bPBM efficacy in PD.

## 5 Conclusions and future directions

Based on therapeutic effects evidenced at the preclinical level, bPBM emerged as a potential future disease-modifying treatment in AD and PD, which are burdensome diseases that sorely lack treatment.

So far, several clinical studies have investigated bPBM therapy, at various parameters, both in patients with AD and associated dementia, and PD. All demonstrate bPBM safety and bring valuable clinical information regarding efficacy, with particularly promising results in AD. However, their exploratory design and inconsistent quality lead to a low level of evidence, which currently does not to support the widespread use of bPBM in clinical practice. The variety of and protocols, the lack of common dosimetry calculation methods and the inherent difficulty to account for individual variability makes comparison between studies difficult.

Future clinical research should address three gaps.

The first gap is the need for robust double-blinded RCTs vs sham, with a higher number of patients and a longer follow-up, to further investigate bPBM efficacy. The ongoing or unpublished clinical studies on bPBM should fill in this gap.

The second gap is the need for research focusing on dosimetry. Monte Carlo simulations of photon travels inside tissues appear as a valuable tool to (i) estimate the range of the dose received by the targeted tissues, (ii) help comparisons between studies and (iii) define optimal bPBM parameters.

The third gap is the need to focus research on imaging and fluid-based biomarkers which are crucial for assessing dose delivery and effects on AD and PD pathophysiology. The lack of biomarkers for assessing PBM effects (pharmacokinetic/pharmacodynamic relationships) is a significant challenge in the field and there is a need for developing and validating appropriate biomarkers for PBM research.

In conclusion, while bPBM shows promise for treating Alzheimer's and Parkinson's diseases, more rigorous clinical research is needed to overcome current challenges and establish its efficacy. Standardized protocols, careful consideration of placebo effects, and high-quality replication studies are crucial for advancing the field.

## Funding

This narrative review has been funded by REGEnLIFE.

## CRediT authorship contribution statement

**Guillaume Blivet:** Writing – review & editing, Writing – original draft, Validation, Supervision, Data curation, Conceptualization. **Benjamin Touchon:** Writing – original draft, Resources, Methodology, Formal analysis, Data curation. **Hugo Cavadore:** Writing – original draft, Methodology, Data curation. **Sara Guillemin:** Writing – original draft, Project administration, Methodology, Formal analysis, Conceptualization. **Frédéric Pain:** Writing – original draft. **Michael Weiner:** Writing – review & editing, Validation, Supervision. **Marwan Sabbagh:** Writing – review & editing, Writing – original draft, Validation, Supervision. **Cécile Moro:** Writing – review & editing, Supervision, Methodology, Conceptualization. **Jacques Touchon:** Writing – review & editing, Writing – original draft, Validation, Supervision.

## Conflict of interest

GB is an employee and owns equity of REGEnLIFE SAS. JT is a consultant for REGENLIFE SAS. SG is an employee of RCTs, who received contract fees from REGEnLIFE SAS for the writing of this review.

FP has no relevant financial interests and no other potential conflicts of interest to disclose.

JT serves on Editorial Board for the Journal for Prevention of Alzheimer’s Disease (JPAD) and is chairman of CTAD Congress and JT Conseil. He has provided consultation to Medesis Pharma, REGEnLIFE, Ariana Pharmaceuticals.

MS has provided consultation to Roche-Genentech, Eisai, Lilly, NeuroTherapia, Signant Health, Novo Nordisk, Prothena, Anavex, Cognito Therapeutics, GSK, Abbvie. MS has stocks/options in OAthira, Lighthouse Pharmaceuticals. MS is a member of EIP Pharma/CervoMed Board of Directors. JC has provided consultation to Acadia, Acumen, ALZpath, Annovis, Aprinoia, Artery, Biogen, Biohaven, BioXcel, Bristol-Myers Squib, Eisai, Fosun, GAP Foundation, Green Valley, Janssen, Karuna, Kinoxis, Lighthouse, Lilly, Lundbeck, LSP/eqt, Merck, MoCA Cognition, New Amsterdam, Novo Nordisk, Optoceutics, Otsuka, Oxford Brain Diagnostics, Praxis, Prothena, ReMYND, Roche, Scottish Brain Sciences, Signant Health, Simcere, Sinaptica, TrueBinding, and Vaxxinity pharmaceutical, assessment, and investment companies. JC owns the copyright of the Neuropsychiatric Inventory. JC has stocks/options in Artery, Vaxxinity, Behrens, Alzheon, MedAvante-Prophase, Acumen. JC is supported by NIGMS grant P20GM109025; NIA grant R35AG71476; NIA R25 AG083721–01; Alzheimer’s Disease Drug Discovery Foundation (ADDF); Ted and Maria Quirk Endowment; Joy Chambers-Grundy Endowment.

MW serves on Editorial Boards for Alzheimer’s & Dementia, and the Journal for Prevention of Alzheimer’s Disease (JPAD). He has served on Advisory Boards for Acumen Pharmaceutical, Alzheon, Inc., Amsterdam UMC; MIRIADE, Cerecin, Merck Sharp & Dohme Corp., NC Registry for Brain Health, and REGEnLIFE. He also serves on the USC ACTC grant which receives funding from Eisai. He has provided consulting to Boxer Capital, LLC, Cerecin, Inc., Clario, Dementia Society of Japan, Dolby Family Ventures, Eisai, Guidepoint, Health and Wellness Partners, Indiana University, LCN Consulting, MEDA Corp., Merck Sharp & Dohme Corp., NC Registry for Brain Health, Prova Education, T3D Therapeutics, University of Southern California (USC), and WebMD. He has acted as a speaker/lecturer for China Association for Alzheimer’s Disease (CAAD) and Taipei Medical University, as well as a speaker/lecturer with academic travel funding provided by: AD/PD Congress, Amsterdam UMC, Cleveland Clinic, CTAD Congress, Foundation of Learning; Health Society (Japan), Kenes, U. Penn, U. Toulouse, Japan Society for Dementia Research, Korean Dementia Society, Merck Sharp & Dohme Corp., National Center for Geriatrics and Gerontology (NCGG; Japan), University of Southern California (USC). He holds stock options with Alzeca, Alzheon, Inc., ALZPath, Inc., and Anven. He received support for his research from the following funding sources: National Institutes of Health (NIH)/NINDS/National Institute on Aging (NIA), Department of Defense (DOD), California Department of Public Health (CDPH), University of Michigan, Siemens, Biogen, Hillblom Foundation, Alzheimer’s Association, Johnson & Johnson, Kevin and Connie Shanahan, GE, VUmc, Australian Catholic University (HBI-BHR), The Stroke Foundation, and the Veterans Administration.
